# Age-Related Trajectories of the Triglyceride–Glucose Index and All-Cause Mortality in Older Chinese Adults

**DOI:** 10.31083/RCM50652

**Published:** 2026-09-16

**Authors:** Chenxi Li, Chenxi Ouyang, Jie Wang, Zuoshi Wen, Han Zhang, Yiwen Dai, Liujun Jiang, Mengjia Chen, Zhangquan Ying, Dongchen Zhou, Biqi Zhang, Yao Lu, Ting Chen

**Affiliations:** ^1^Department of Cardiology, The First Affiliated Hospital, College of Medicine, Zhejiang University, 310003 Hangzhou, Zhejiang, China; ^2^Clinical Research Center, The Third Xiangya Hospital, Central South University, 410013 Changsha, Hunan, China; ^3^Faculty of Life Sciences & Medicine, King’s College London, SE1 9NH London, UK

**Keywords:** triglyceride–glucose index, insulin resistance, all-cause mortality, longitudinal trajectories, cardiovascular disease

## Abstract

**Background:**

The triglyceride–glucose (TyG) index is recognized as an alternative biomarker of cardiometabolic health, associated with insulin resistance and the development and prognosis of cardiovascular disease (CVD). However, the prospective association between long-term TyG index trajectories and all-cause mortality in the older population remains incompletely elucidated.

**Methods:**

This longitudinal retrospective cohort study included 93,418 older adults who underwent repeated general health assessments at The Third Xiangya Hospital of Central South University between 2012 and 2020. The TyG index was calculated utilizing the formula: ln (fasting triglycerides (mg/dL) × fasting glucose (mg/dL)/2). TyG index trajectories during the follow-up were analyzed using latent class trajectory modeling (LCTM). Baseline TyG index values and TyG trajectories were analyzed using a Cox proportional hazards model to estimate hazard ratios (HRs) and 95% confidence intervals (CIs).

**Results:**

Over a median follow-up of 5.4 years, 7364 participants died, including 4176 cardiovascular deaths. Each 1-standard deviation (SD) increase in the TyG index was associated with a 18.9% higher risk of all-cause mortality, even after accounting for traditional CVD risk factors (HR 1.189, 95% CI 1.144–1.235). Similar associations were observed when the TyG index was modeled in quartiles. Based on the observed trajectory patterns, participants were classified into four groups: low, moderate–low, moderate, and high TyG index trajectories. The low, moderate–low, and high trajectory groups were independently associated with all-cause mortality, whereas the moderate group was not. Following multivariable adjustment, the moderate–low group exhibited a 1.104-fold increase in all-cause mortality risk (95% CI: 1.047–1.165), whereas the high group demonstrated a 1.243-fold increase (95% CI: 1.139–1.357), with both findings statistically significant (*p* < 0.001). Meanwhile, no significant association was found between the low group and the study outcomes (HR 0.998, 95% CI 0.875–1.138; *p* = 0.972).

**Conclusions:**

In this large cohort of older adults, moderate-low and high TyG index trajectories were associated with an increased risk of all-cause mortality. Longitudinal patterns of the TyG index may serve as important indicators for identifying individuals at elevated risk of all-cause mortality and may support the development of targeted preventive and therapeutic interventions.

## 1. Introduction

Research has demonstrated a significant connection between insulin resistance (IR) and cardiovascular diseases (CVDs), emphasizing the important role of metabolic disorders in the onset and progression of these conditions [1,2,3]. The triglyceride–glucose (TyG) index serves as a straightforward, accessible, and reliable clinical surrogate marker for assessing IR [4]. A substantial body of research has rigorously examined the association between the TyG index and a range of diseases, with a particular focus on metabolic disorders [5,6] and cardiovascular conditions [7,8,9,10,11]. Nevertheless, research in the general population has yielded mixed results regarding the association between the TyG index and all-cause mortality (ACM) [12,13,14] and cardiovascular mortality (CVM) [15,16,17].

However, with advancing age, IR tends to increase owing to changes in body fat distribution, lipid metabolism, and insulin signaling [18]. Therefore, continuous monitoring of the TyG index is essential. Fluctuations in glucose and increasing plasma triglyceride levels, both associated with CVD risk, highlight the importance of considering these time-related changes [19,20]. Most previous studies have assessed the TyG index at a single time point, with comparatively few investigations into the fluctuations and longitudinal trajectories of the index. A prospective investigation of the Coronary Artery Risk Development in Young Adults (CARDIA) study, which included participants aged 18–30 years, found that each 1-unit increase in the TyG index was associated with an 85% increase in the risk of death in young, healthy adults. Furthermore, persistently high TyG index levels in early adulthood were significantly associated with increased risks of CVD events and ACM in subsequent years [21]. Moreover, a large prospective cohort study in older adults demonstrated that participants exhibiting trajectories characterized by moderate stability or a high, gradual increase in the TyG index had a higher risk of CVDs than those who maintained low levels [22]. Despite these findings, the effects of TyG index fluctuations and trajectories on all-cause mortality and on mortality unrelated to cardiovascular and cerebrovascular conditions in older adults remain poorly understood. Findings from a cohort study involving Iranian adults indicated a substantial direct connection between the TyG index and mortality rates for both CVD and ACM. However, the predictive utility of the TyG index for mortality outcomes might be influenced by diabetes status, and no significant association was observed between the TyG index and non-cardiovascular mortality [15]. Consequently, these findings suggest that long-term regulation of blood glucose and lipid levels in older people could significantly affect mortality outcomes.

Building on the aforementioned context, the present study aimed to investigate and substantiate the potential association between long-term fluctuations in the TyG index and ACM in an older population. Thus, this analysis was conducted using data from an extensive, single-center longitudinal cohort of Chinese older adults. The findings of this research are expected to provide important insights for informing public health strategies and clinical approaches targeting the older population.

## 2. Methods

### 2.1 Study Population

This study included urban Chinese residents who underwent annual physical examinations at The Third Xiangya Hospital of Central South University from 2012 to 2020. Most participants were from Hunan Province, Jiangxi Province, and other regions in southern China. The initial physical examination records were used as baseline data. The following exclusion criteria were applied: (1) aged <65 years; (2) fewer than three follow-up visits; (3) missing records of fasting blood glucose (FBG) and triglycerides (TGs); (4) missing lifestyle records, including smoking, drinking, and exercise (n = 200,774); (5) missing records of blood pressure, blood glucose, and blood lipids (n = 358,122).The final cohort comprised 93,418 participants (Fig. 1). **Supplementary Table 1** compares the final cohort with the original total population. Most baseline covariates (age, sex, lifestyle, etc.) showed good balance (standardized mean difference (SMD) <0.2), except for body mass index (BMI) (SMD = 0.256), which was slightly higher in the original population, indicating that a higher BMI was associated with missingness.

**Fig. 1.**
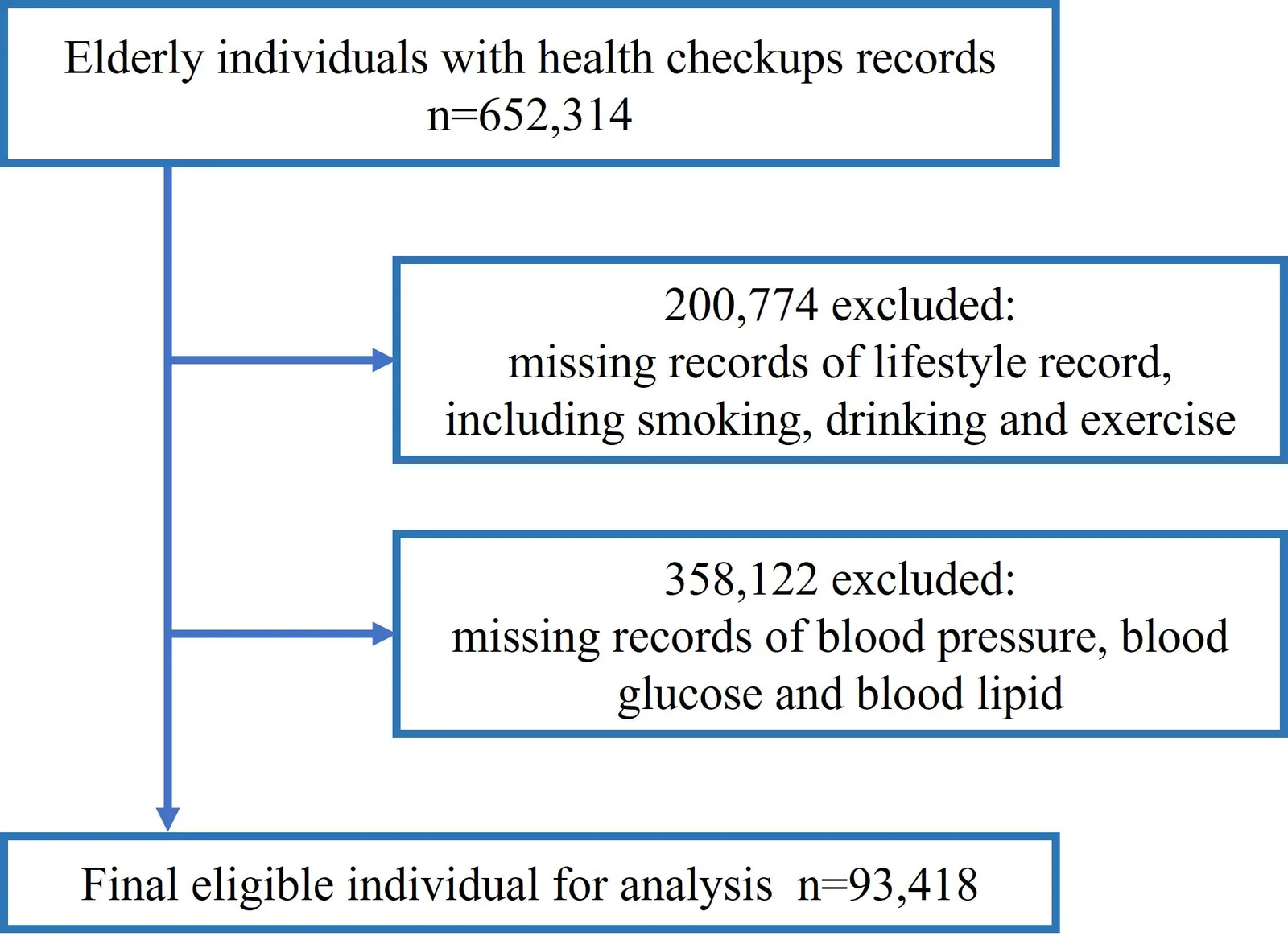
**Study cohort flow diagram**.

Informed consent was obtained from all participants before study enrolment. The study was conducted in accordance with the Declaration of Helsinki and was approved by the Institutional Review Board of The Third Xiangya Hospital located in Changsha, China.

### 2.2 Data Collection

A standardized questionnaire was used to collect baseline data on demographics, health habits, medical history, and current medication use. Smoking status was categorized as ‘current’ (used tobacco within the last 6 months or quit within that period), ‘former’ (abstained from smoking or drinking for more than 6 months), or ‘never’. Drinking was defined as ‘everyday’, ‘occasionally’, ‘always’, or ‘never’. Exercise routines were categorized based on weekly frequency (‘everyday’, ‘at least once a week’, ‘occasionally’, or ‘never’), with the first category indicating regular physical activity. Trained personnel performed all anthropometric measurements. The height and weight of each participant were recorded to the nearest 0.1 cm and 0.1 kg, respectively, while participants wore light indoor clothing and no shoes. BMI was calculated as weight (kg)/height (m)^2^. Waist circumference (WC) was measured at the midpoint between the costal margin and the iliac crest. Blood pressure (BP) was assessed in a seated position in accordance with the guidelines outlined in the Seventh Report of the Joint National Committee (JNC 7). Measurements were recorded at 5-minute intervals on three separate occasions, and the mean value was subsequently calculated. In cases where the difference between any two readings exceeded 10 mmHg, the two measurements with the smallest difference were used for analysis. Serum FBG, TG, total cholesterol (TC), low-density lipoprotein cholesterol (LDL-C), high-density lipoprotein cholesterol (HDL-C), and serum creatinine (Scr) were measured using an automated analyzer (Hitachi 7600–110; Hitachi, Tokyo, Japan). The TyG index was calculated using the formula: ln (fasting triglyceride (mg/dL) × fasting glucose (mg/dL)/2).

### 2.3 Definitions of Hypertension, Diabetes, and Dyslipidemia

Hypertension was defined as a systolic blood pressure (SBP) ≥140 mmHg, a diastolic blood pressure (DBP) ≥90 mmHg, or use of antihypertensive medication. Diabetes was defined as an FBG ≥7.0 mmol/L, a 2-hour postprandial blood glucose ≥11.1 mmol/L, or use of insulin or oral hypoglycemic agents. Dyslipidemia was defined as LDL-C ≥4.1 mmol/L, TC ≥6.2 mmol/L, TG ≥2.3 mmol/L, or HDL-C <1.0 mmol/L, in accordance with the Chinese Guideline for Lipid Management (2023).

### 2.4 Study Outcome

The primary outcome was ACM, while the secondary outcomes included cardiovascular and non-CVM. Mortality data were collected from the National Mortality Surveillance System (NMSS), administered by the Centers for Disease Control and Prevention in Hunan Province. Residents are linked to the NMSS through their health record identification numbers, which provide data on time and cause of death, as well as the corresponding ICD-10 classification. Deaths with cardiovascular diagnoses (ICD-10 codes I00–I90) were classified as CVM; deaths from all other causes were classified as non-CVM.

### 2.5 Statistical Analysis

All analyses were conducted using Stata version 17.0 (StataCorp LLC, College Station, TX, USA). TyG trajectories were identified using latent class trajectory modeling (LCTM) implemented with the user-written ‘traj’ procedure in Stata. Using a finite mixture modeling approach, we summarized the complex longitudinal course of the TyG index into distinct trajectory classes, thereby identifying subgroups of participants with similar patterns over time. To capture age-related changes in the TyG index and account for the irregular timing of health examination visits, we used age rather than calendar time as the time axis in LCTM. This approach follows the methodological framework proposed by Lennon et al. [23], in which longitudinal data are aligned on the age scale to model developmental trajectories across the life course. Specifically, each TyG measurement was aligned to the age of the participant at the time of measurement. The resulting dataset contained multiple age-aligned observations per participant, with ages ranging from 65 to 105 years. For each participant, the first available TyG measurement was assigned to their chronological age at that visit, and subsequent measurements were incorporated according to the age of the participant at the time of measurement. All LCTMs were fitted using age-aligned TyG values as the time-varying outcome. A censored normal distribution was specified for the TyG index. The number and shape (intercept-only, linear, quadratic, or cubic) of TyG trajectories were selected based on the following criteria: minimization of the Bayesian information criterion (BIC), high average posterior probabilities (APPA) (>0.7), and at least 5% of the total sample in each trajectory group (**Supplementary Table 2**). However, the final model included one group with 3.1% of participants, as this solution provided the best overall fit and clinical interpretability. Model adequacy was further assessed using discrimination tools (degree of separation and examination of residual plots) and by evaluating the clinical plausibility of the trajectory classes. Model discriminative validity was evaluated using two commonly applied approaches. First, the APPA for each trajectory group were all above 0.7 (Group 1: 0.762; Group 2: 0.776; Group 3: 0.837; Group 4: 0.851), indicating excellent classification accuracy. Second, the Elsensohn envelope of residuals shows clear separation among the four trajectory groups across most ages (**Supplementary Fig. 1**). Collectively, these results confirm that the identified trajectory classes are well separated and statistically robust. Following a systematic selection procedure, we identified the model with four linear trajectories as the best fit. Detailed step-by-step calculations are provided in the **Supplementary Materials**. Missing TyG values at the individual-observation level were handled by a complete case analysis, as participants with incomplete data at any visit had already been excluded during cohort construction; the potential impact of this approach on trajectory estimates is addressed in the Discussion section. The characteristics of the TyG index trajectory groups were compared using analysis of variance (ANOVA) or Kruskal–Wallis H-tests for continuous variables and chi-squared tests for categorical variables. To investigate the association between trajectory classes and mortality, we applied a Cox proportional hazards regression model, using follow-up time as the time scale. All potential covariates in the regression models were measured at baseline, including age, sex, smoking, drinking, exercise, BMI, WC, serum creatinine, estimated glomerular filtration rate (eGFR), hypertension, and dyslipidemia. Our study comprised three models: Model 1 adjusted for age and sex. Model 2 further adjusted for smoking, drinking, and exercise. Model 3 further adjusted for BMI, WC, serum creatinine, eGFR, SBP, DBP, TC, LDL-C, and HDL-C. Model 4 further adjusted for BMI, WC, serum creatinine, eGFR, hypertension, and dyslipidemia, based on Model 2. In the longitudinal analysis, baseline characteristics by TyG index quartiles were summarized as the mean with standard deviation (SD), median with interquartile range (IQR), or frequencies with percentages (%), as appropriate. Continuous variables were analyzed using the Mann–Whitney U test for two independent samples or the Kruskal–Wallis H-test for more than two independent samples. In contrast, categorical variables were examined using the chi-squared test or Fisher’s exact test. The association between baseline TyG index quartiles, TyG index per standard deviation change, and all endpoints was examined utilizing the Cox proportional hazards regression model. The non-linear relationship between TyG and mortality risk was assessed using a restricted cubic spline (RCS) function within a Cox proportional hazards model. The dose–response relationships between TyG and all endpoints were explored using 4 internal knots in TyG distributions within the RCS.

To evaluate the robustness of our findings, we conducted multiple sensitivity analyses. First, participants who died within the first two years were excluded from the study to mitigate the potential for reverse causality, as a brief follow-up period could influence the results. Second, to mitigate the influence of pharmacological interventions, we excluded participants taking antidiabetic medications to ascertain whether the results were affected. Third, we excluded participants with FBG ≥7.0 mmol/L and TG ≥1.7 mmol/L at baseline. Furthermore, to attain a comprehensive understanding of the potential relationship between changes in the TyG index and mortality risk, subgroup analyses were performed by sex (male vs. female), BMI (<28 vs. ≥28 kg/m^2^), and presence of diabetes mellitus. These analyses evaluated whether the association between TyG trajectories and mortality differed across these subgroups.

Statistical significance was defined as a two-sided *p*-value <0.001, and the proportional hazards assumption was satisfied for all Cox proportional hazards models. All statistical analyses were conducted using Stata version 17.0 (StataCorp, College Station, TX, USA) and R version 4.4.3 (R Core Team, 2024, Vienna, Austria).

## 3. Results

### 3.1 Population Characteristics

The study included 93,418 participants, among whom 7364 deaths were documented. Of these, 4176 were attributed to cardiovascular causes, and 3188 were related to cancer, over a median follow-up of 5.4 years (range, 2–9 years). The median age was 71 years, and 32,435 (34.72%) participants were male. Among all older individuals, 70,077 (75.01%) had hypertension, and 47,291 (50.62%) had diabetes.

### 3.2 Baseline Characteristics and Association With Mortality According to TyG Index Quartiles

The median TyG index at baseline was 7.39. Baseline characteristics according to TyG index quartiles are presented in the Additional file: **Supplementary Table 2**. Participants in higher TyG quartiles tended to have higher BMI, WC, SBP, DBP, FBG, TC, TG, and LDL-C levels; a higher prevalence of hypertension, diabetes, and dyslipidemia; more frequent use of antihypertensive and hypoglycemic medications, as well as lower HDL-C levels compared with those in lower TyG quartiles. An elevated TyG index was associated with greater cardiometabolic risk, and the risk of ACM increased across higher TyG quartiles. After comprehensive adjustments for potential confounders, a 1-SD increase in the TyG index was associated with a 18.9% increase in the risk of ACM in the multivariable model that evaluated the TyG index as a continuous variable (HR 1.189, 95% CI 1.144–1.235; *p* = 0.000; **Supplementary Table 3**). Comparable outcomes were noted when individuals were grouped by TyG index quartiles; notably, those in the highest TyG index quartile had the greatest risk of ACM across all three adjusted models (all *p* < 0.05; **Supplementary Table 4**; **Supplementary Fig. 2**).

Fig. 2 shows the Kaplan–Meier survival curves for ACM, categorized by the quartiles of the baseline TyG index (log-rank test, *p* < 0.05). In the Kaplan–Meier curve, the third TyG quartile (Q3) shows the highest long-term survival, which is consistent with the trajectory patterns observed in this study. The dose–response relationship between TyG and all endpoints was explored using four internal knots in TyG distributions within the restricted cubic spline (RCS) model. The RCS curve shows a reverse L-shaped curve (Fig. 3). The results indicate a significant correlation between the baseline TyG index and ACM.

**Fig. 2.**
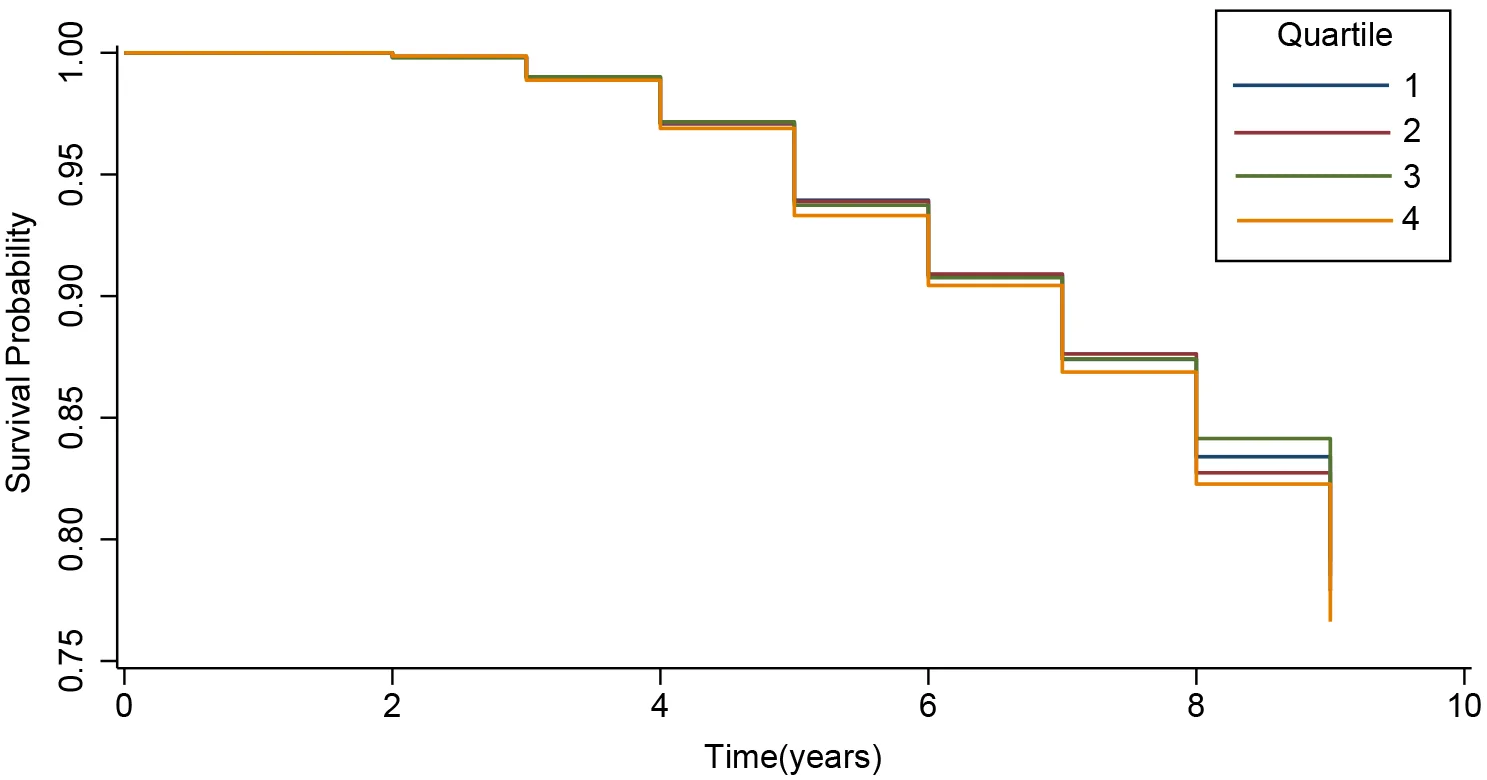
**Kaplan–Meier survival curves for all-cause mortality by baseline TyG index quartiles**.

**Fig. 3.**
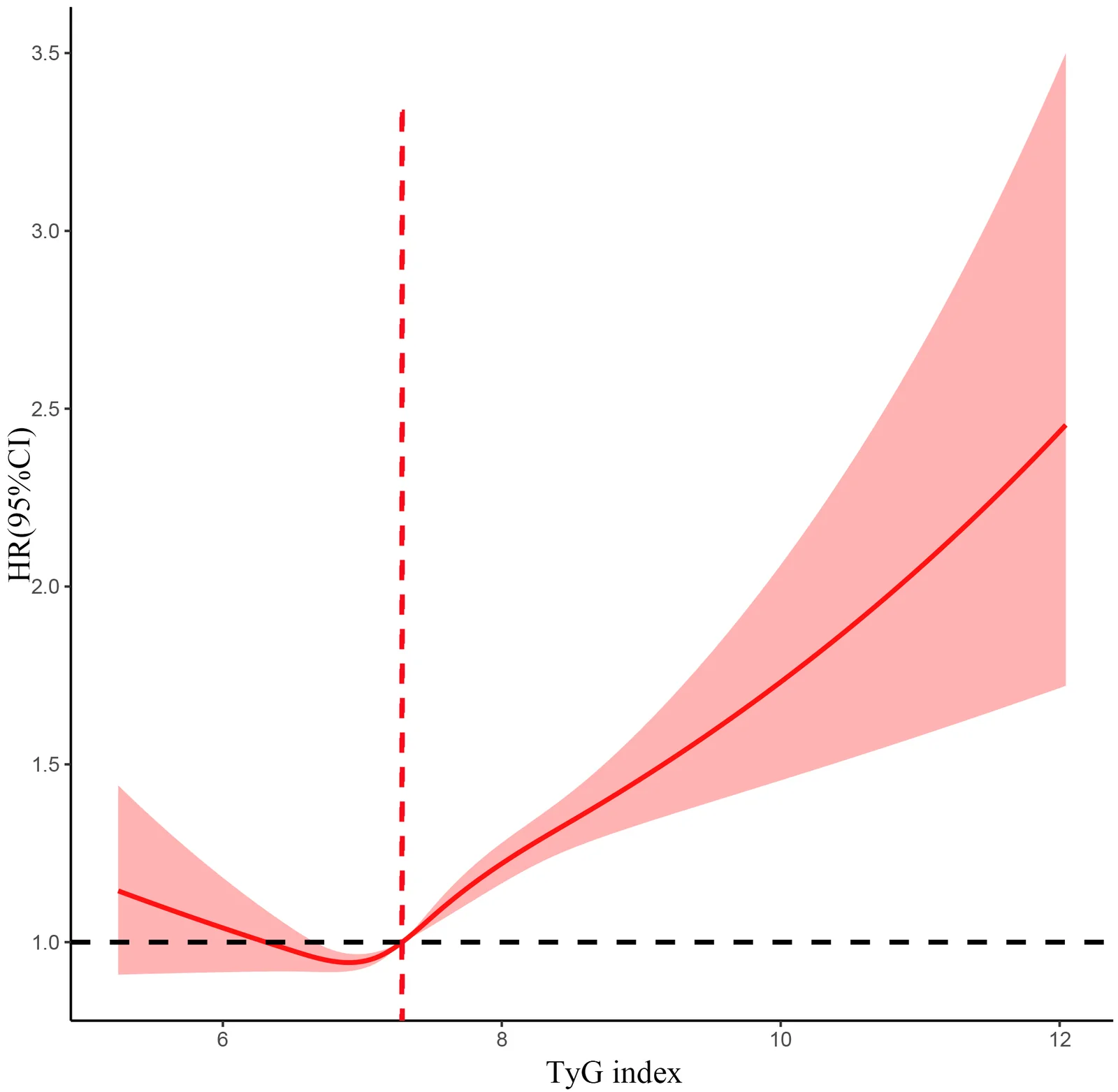
**Adjusted dose–response relationships between the triglyceride–glucose (TyG) index and clinical outcomes**.

### 3.3 Baseline Characteristics of Study Participants by Distinct TyG Trajectories

The trajectory analysis included 451,540 participants. Based on model adequacy criteria and interpretability rules, four distinct TyG index trajectories were identified. Fig. 4 illustrates the final latent class trajectory model for the TyG index, comprising four groups: low (12.6%), moderate–low (64.8%), moderate (19.5%), and high (3.1%).

**Fig. 4.**
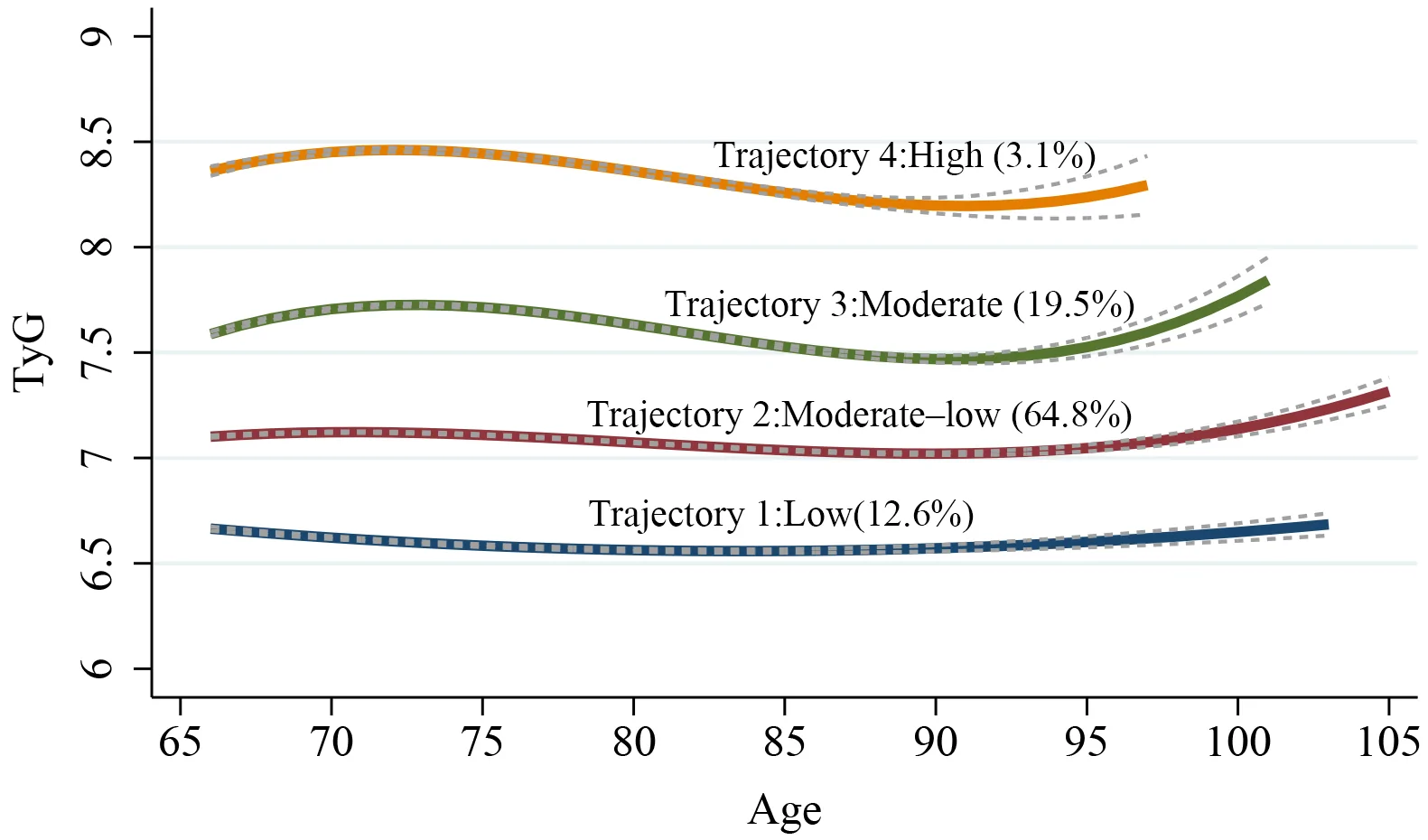
**Distribution of participants across TyG index trajectory groups and their respective percentages**.

Table 1 presents the baseline demographic and clinical characteristics according to TyG index trajectories. Individuals in the high TyG trajectory group exhibit a higher prevalence of hypertension, diabetes, and dyslipidemia, as well as increased BMI, WC, FBG, TG, and LDL-C, and lower HDL-C and eGFR levels. In addition, baseline use of antihypertensive medications differed significantly among the TyG trajectory groups, whereas serum creatinine levels did not differ significantly across trajectories.

**Table 1.** **Baseline characteristics of participants in different TyG index trajectories**.

Characteristics		Total	Trajectory 1	Trajectory 2	Trajectory 3	Trajectory 4	*p*-value
Age, years		71.35 ± 5.07	71.71 ± 5.19	71.60 ± 5.16	71.05 ± 4.89	70.78 ± 4.99	<0.001
Male, n (%)		32,435 (34.72)	1633 (52.12)	19,340 (37.38)	9234 (30.31)	2228 (27.55)	<0.001
Smoking, n (%)							<0.001
	1 (never)	82,276 (88.07)	2602 (83.05)	45,326 (87.61)	27,123 (89.04)	7225 (89.34)	
	2 (former)	2477 (2.65)	98 (3.13)	1436 (2.78)	763 (2.5)	180 (2.23)	
	3 (current)	8665 (9.28)	433 (13.82)	4975 (9.62)	2575 (8.45)	682 (8.43)	
Drinking, n (%)							<0.001
	1 (never)	86,023 (92.08)	2744 (87.58)	47,418 (91.65)	28,318 (92.96)	7543 (93.27)	
	2 (occasionally)	4329 (4.63)	205 (6.54)	2503 (4.84)	1306 (4.29)	315 (3.9)	
	3 (always)	1252 (1.34)	61 (1.95)	804 (1.55)	316 (1.04)	71 (0.88)	
	4 (everyday)	1814 (1.94)	123 (3.93)	1012 (1.96)	521 (1.71)	158 (1.95)	
Regular exercise, n (%)							<0.001
	1 (everyday)	24,351 (26.13)	884 (28.28)	12,726 (24.66)	8427 (27.71)	2314 (28.71)	
	2 (≥1 times a week)	4712 (5.06)	146 (4.67)	2587 (5.01)	1584 (5.21)	395 (4.9)	
	3 (occasionally)	10,372 (11.13)	333 (10.65)	5855 (11.34)	3343 (10.99)	841 (10.43)	
	4 (never)	53,774 (57.69)	1763 (56.4)	30,445 (58.99)	17,055 (56.09)	4511 (55.96)	
BMI, kg/m^2^		24.06 ± 3.09	22.86 (2.95)	23.69 (3.04)	24.59 (3.07)	24.90 (3.03)	<0.001
WC, cm		82.35 ± 9.41	79.52 ± 9.16	81.37 ± 9.29	83.66 (9.35)	84.78 (9.33)	<0.001
SBP, mmHg		138.25 ± 17.00	136.60 ± 16.75	137.01 ± 16.48	139.70 (17.43)	141.33 (17.90)	<0.001
DBP, mmHg		81.72 ± 9.27	80.86 ± 9.65	81.31 ± 9.04	82.16 ± 9.43	83.01 ± 9.73	<0.001
Fasting glucose, mmol/L		6.77 ± 2.62	6.03 ± 2.08	6.39 ± 2.36	7.15 ± 2.72	8.08 ± 3.25	<0.001
TC, mmol/L		4.79 ± 3.10	4.58 ± 3.37	4.73 ± 3.53	4.84 ± 2.45	5.06 ± 2.09	<0.001
TG, mmol/L		1.87 ± 1.97	1.33 ± 1.79	1.64 ± 2.00	2.02 ± 1.62	2.98 ± 2.47	<0.001
LDL-C, mmol/L		2.57 ± 0.91	2.43 ± 0.82	2.53 ± 0.88	2.64 ± 0.95	2.64 ± 1.00	<0.001
HDL-C, mmol/L		1.50 ± 0.83	1.54 ± 0.78	1.52 ± 0.85	1.47 ± 0.86	1.41 ± 0.68	<0.001
Serum creatinine, μmol/L		82.57 ± 25.55	83.08 ± 26.00	82.42 ± 25.09	82.68 ± 25.94	82.88 ± 26.75	>0.05
eGFR, mL/min/1.73 m^2^		91.45 ± 155.74	98.93 ± 188.54	92.68 ± 157.25	89.57 ± 155.60	87.74 ± 130.28	<0.001
Hypertension, n (%)		70,077 (75.01)	2219 (70.83)	37,479 (72.44)	23,812 (78.17)	6567 (81.2)	<0.001
Diabetes, n (%)		47,291 (50.62)	1208 (38.56)	22,975 (44.41)	17,525 (57.53)	5583 (69.04)	<0.001
Dyslipidemia, n (%)		27,806 (29.77)	517 (16.5)	11,032 (21.32)	11,517 (37.81)	4740 (58.61)	<0.001
Anti-hypertensive medication, n (%)		65,638 (70.26)	2036 (64.99)	35,999 (69.58)	21,838 (71.69)	5765 (71.29)	<0.001
Anti-hyperglycemia medication, n (%)		12,839 (13.74)	382 (12.19)	6568 (12.69)	4529 (14.87)	1360 (16.82)	<0.001

BMI, body mass index; WC, waist circumference; SBP, systolic blood pressure; DBP, diastolic blood pressure; TC, total cholesterol; TG, triglycerides; LDL-C, low-density lipoprotein cholesterol; HDL-C, high-density lipoprotein cholesterol; eGFR, estimated glomerular filtration rate.

### 3.4 Association Between Distinct TyG Trajectories and All-Cause Mortality

Consistent with previous studies indicating that intermediate TyG levels are more favorable for the health of older adults, we selected Trajectory 3 as the reference group for comparisons with the other three trajectories (**Supplementary Table 5**). Fig. 5 depicts the associations between TyG index trajectories and ACM. Notably, ACM was independently associated with the low, moderate-low, and high-increasing groups compared with the moderate group. After adjusting for age, sex, smoking, drinking, exercise, BMI, WC, serum creatinine, eGFR, hypertension, and dyslipidemia, the moderate-low group had a 1.104-fold (95% CI 1.047–1.165) risk of ACM, while the high group had a 1.243-fold (95% CI 1.139–1.357) risk of ACM (both *p* < 0.001). In contrast, there was no significant association in the low group (HR 0.998; 95% CI 0.875–1.138; *p* = 0.972).

**Fig. 5.**
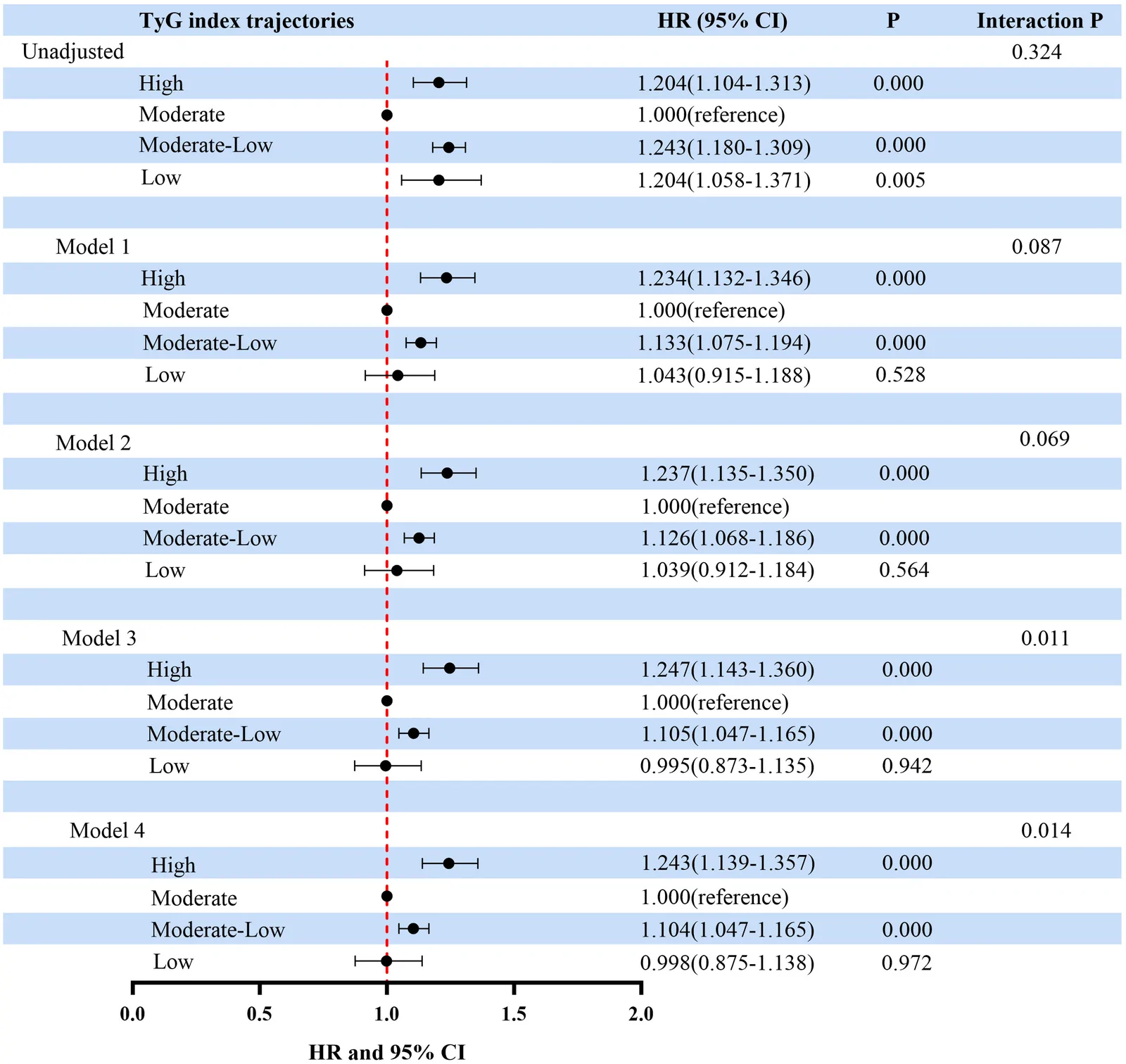
**Hazard ratios (HRs) and 95% confidence intervals (CIs) for all-cause mortality by trajectory groups of the TyG index**. Model 1: Adjusted for age and sex. Model 2: Adjusted for age, sex, smoking status, alcohol consumption, and exercise. Model 3: Adjusted for age, sex, smoking status, alcohol consumption, exercise, BMI, WC, serum creatinine, eGFR, SBP, DBP, TC, LDL-C, and HDL-C. Model 4: Adjusted for age, sex, smoking status, alcohol consumption, exercise, BMI, WC, serum creatinine, eGFR, hypertension, and dyslipidemia.

### 3.5 Sensitivity Analyses

A sensitivity analysis was performed to assess the robustness of the findings. First, we excluded participants who died within the first two years of follow-up. Second, we excluded participants who used any antidiabetic drugs to evaluate whether medication use influenced the outcomes. Third, we separately excluded participants with baseline FBG ≥7.0 mmol/L and those with TG ≥1.7 mmol/L. The results of these analyses were consistent with those of the primary analysis (**Supplementary Table 6**). In addition, there was no significant difference in cardiovascular or other mortality outcomes, regardless of whether competing risk analysis was applied (Fig. 6).

**Fig. 6.**
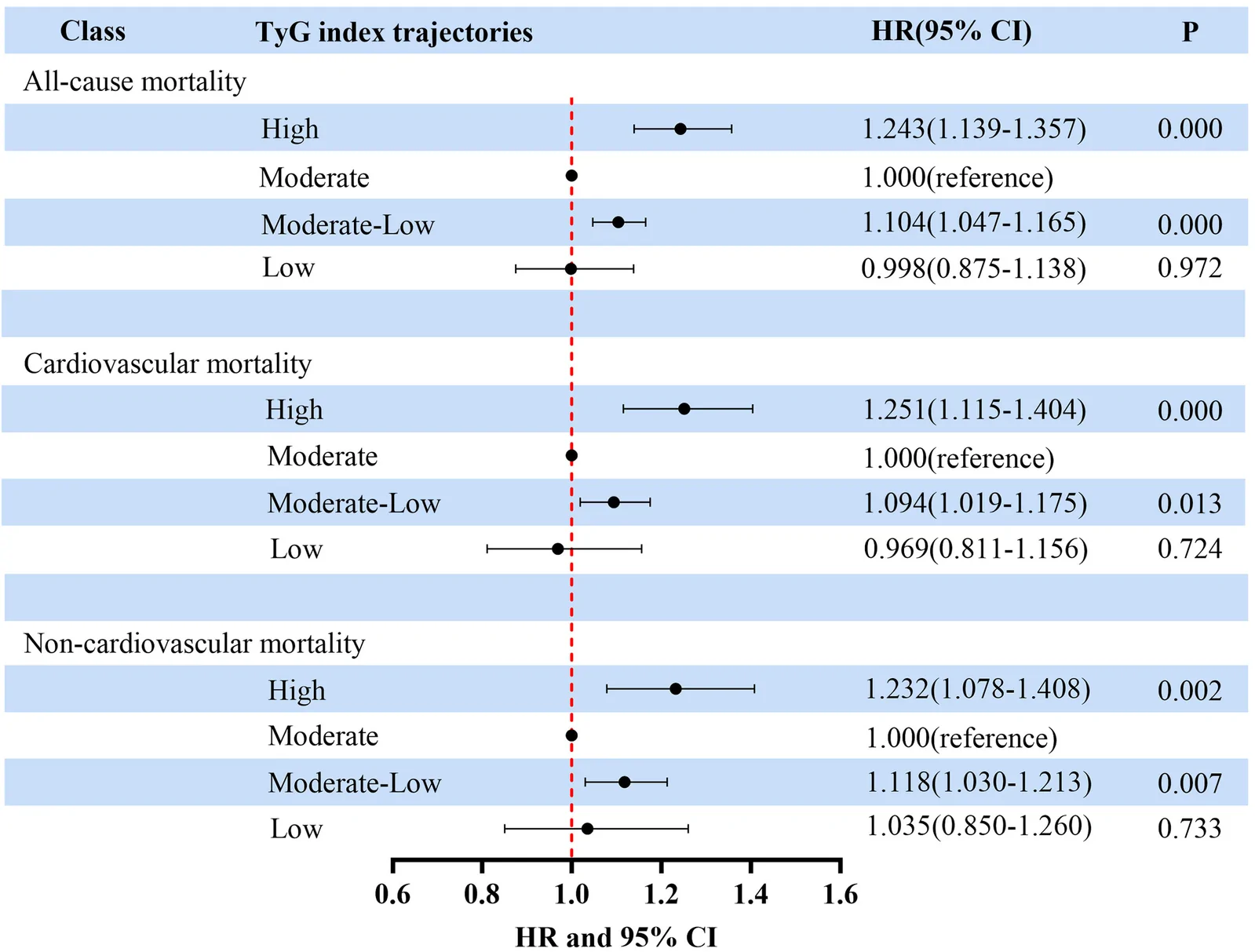
**Adjusted HRs for cause-specific mortality across different trajectories**. Sensitivity analysis 1: Exclusion of deaths within the first 2 years (Model 3). Sensitivity analysis 2: Exclusion of participants using hypoglycemic medications. Sensitivity analysis 3: Exclusion of participants with baseline FBG ≥7.0 mmol/L and TG ≥1.7 mmol/L.

### 3.6 Stratified Analysis

Assessing the potential effect modification by sex, BMI, and history of diabetes, Fig. 7 illustrates the mortality risk for participants categorized by sex (male or female), BMI (<28 or ≥28 kg/m^2^), and diabetes status (yes or no), across distinct TyG trajectories. For males, the hazard ratios for outcome events in the low, moderate–low, and high trajectory groups were 0.895 (95% CI 0.751–1.067), 0.927 (95% CI 0.854–1.006), and 1.044 (95% CI 0.913–1.194), respectively, with all *p*-values > 0.001, *i*.*e*., not statistically significant. Conversely, a higher risk was observed in the low (HR 1.000; 95% CI 0.832–1.202; *p* = 0.999), moderate–low (HR 1.101; 95% CI 1.025–1.181; *p* = 0.008), and high (HR 1.296; 95% CI 1.157–1.452; *p* < 0.001) trajectory groups for females. Similar patterns were observed in the participant with BMI <28 kg/m^2^ and no diabetes (Fig. 7).

**Fig. 7.**
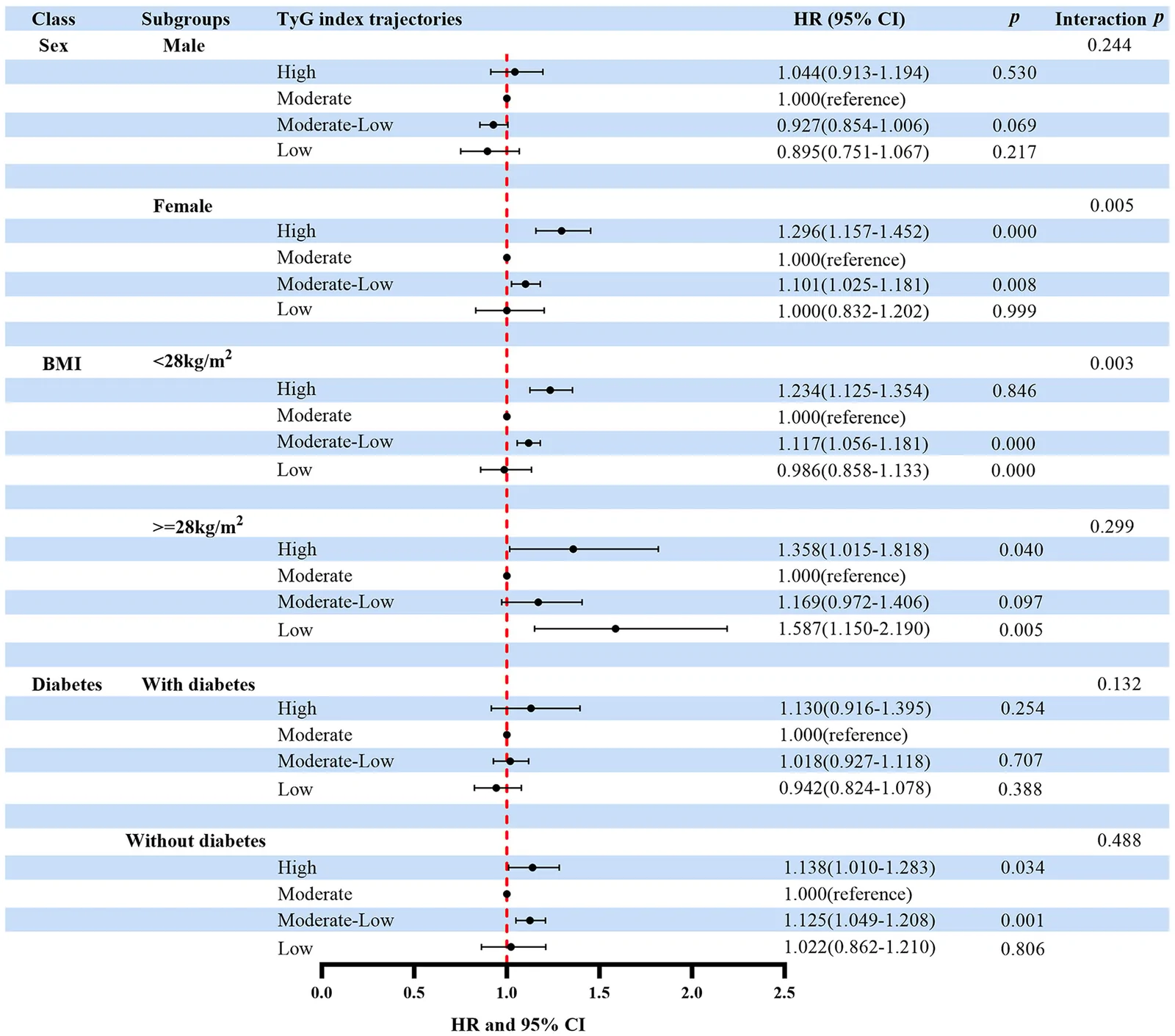
**Adjusted HR for all-cause mortality across different trajectories in stratified analyses**.

## 4. Discussion

This study identified a substantial correlation between TyG index trajectories and both ACM and CVM in the older population, highlighting the potential longitudinal TyG patterns as a predictive tool for mortality. Four separate trajectories of the TyG index were identified, each connected with varying risks of ACM: low-stable, moderate–low-stable, moderate-stable, and high-stable. Trajectory classes were labeled descriptively based on both their baseline level and temporal evolution. The clinical relevance of these labels derives from their correspondence to known stages of IR progression: a low-stable trajectory represents consistent metabolic health, whereas a high-stable or increasing trajectory signifies persistent or worsening IR, a recognized key risk factor for cardiovascular and metabolic diseases. A notable association was similarly observed between the trajectories of the TyG index and ACM rates. Compared with the moderate-stable trajectory, the moderate-low-stable and high-stable trajectories had a higher risk of ACM, whereas the low-stable trajectory showed no significant statistical difference. Moreover, this relationship remained consistent across various subgroups and sensitivity analyses. Our findings indicate that both the level and longitudinal pattern of the TyG index are associated with cardio-cerebral events in older adults. Given that the TyG index is a simple, low-cost surrogate of IR, our results suggest that higher TyG levels and adverse TyG trajectories are associated with increased ACM and CVM among older adults.

Previous research has shown that individuals with an elevated baseline TyG index, particularly when accompanied by a moderately stable or progressively increasing trajectory, have an increased risk of CVD in the older population [22]. A study conducted in 2026 demonstrated that elevated TyG levels were correlated with a higher one-year mortality rate among older patients with acute decompensated heart failure (ADHF) [24]. The trajectories of the TyG index may help identify older individuals at elevated risk of frailty, thereby warranting early preventive and therapeutic interventions [25]. Furthermore, the TyG index has been shown to provide supplementary prognostic information in addition to that of conventional clinical models [26]. This association is consistent among healthy adults of normal weight [27] as well as in individuals diagnosed with type 2 diabetes [28]. In a recent comprehensive prospective cohort study led by Professor Patricio Lopez-Jaramillo [8], involving participants aged 35–70 years, those in the highest TyG tertile had an increased risk of cardiovascular death, whereas no significant association was found between the TyG index and non-CVM. While prior investigations have reported a connection between elevated TyG index levels and an increased risk of ACM in individuals under 65 years [29,30], it is important to recognize that these studies primarily focused on patients with cardiovascular or cerebrovascular diseases, suggesting that ACM outcomes were fundamentally affected by these specific diseases. Consequently, the proportion of cardiovascular deaths was not clearly delineated, and those findings did not fully characterize the relationship between the TyG index and ACM in older adults.

Our study employed a large cohort with a long follow-up period, enabling a thorough investigation of the relationship between TyG index trajectories and mortality. By stratifying participants into categories based on low, moderate–low, moderate, and high TyG index trajectory groups, we gained a clearer understanding of the dynamic nature of IR and its association with mortality risk. This strategy addresses the weaknesses of past studies that relied solely on baseline IR data and single-point measurements [31,32]. Human resistance to insulin-induced cellular stress reduces with advancing age [18], emphasizing the importance of monitoring the TyG index over time. Fluctuations in glucose levels and rising plasma TG levels, which are also linked to CVD risk, highlight the importance of longitudinal assessment. Previous studies have suggested an optimal TyG level associated with the lowest mortality risk [33,34], and our findings further support that long-term maintenance of TyG near this level is beneficial for reducing both CVD and all-cause mortality. Consistent results have been reported in other populations. For example, in patients with type 2 diabetes mellitus (T2DM), baseline TyG and its progression were connected to major cardiovascular complications [35]. In a younger group, research by Xu et al. [21] identified three distinct TyG trajectories (low, moderate, and high), which provided valuable information on cardiovascular risk. By examining fluctuations in the TyG index, we gained a deeper understanding of how these alterations affect long-term mortality from cardiovascular and all other causes. This understanding, in turn, contributes to categorizing risk levels and creating better intervention methods. Additionally, some studies have found no significant association between the TyG index trajectory and adverse CVD outcomes. A study examining the association between the TyG index and carotid atherosclerosis demonstrated that a higher baseline TyG index was significantly associated with the progression of carotid atherosclerosis. Additionally, a moderately stable TyG trajectory was also significantly associated with the progression of carotid atherosclerosis. Nevertheless, no significant relationship was observed between the progression of carotid atherosclerosis and the highly stable TyG index group [36]. The discrepancy observed between the trajectory and baseline results may be attributed to additional factors during long-term follow-up that potentially outweigh the independent effect of the TyG trajectory. Thus, further investigations are required to elucidate the specific underlying causes.

Subgroup analysis showed that ACM risk was elevated in females, individuals with BMI <28 kg/m^2^, and individuals without diabetes. Similar findings have been observed in other studies [37]. This disparity may be ascribed to nutritional risk factors. Given the positive association between the TyG index and fasting TG and glucose levels, individuals with a low TyG index may be at risk of undernutrition. Furthermore, recent reports from China indicate that females exhibit lower BMI and visceral adiposity index (VAI) values than their male counterparts [38,39]. Therefore, in populations at elevated risk of being underweight or malnourished, particularly among females with a BMI <28 kg/m^2^, the potential for nutritional deficiencies may present a greater concern than the metabolic risks associated with IR. In such cases, a moderately elevated TyG index, even if below the established cut-off value, could be associated with improved survival outcomes. These findings underscore the importance of monitoring changes in the TyG index, especially for those in high-risk categories. Continuous monitoring of variations in the TyG index may provide a more reliable assessment than tracking changes in homeostatic model assessment of insulin resistance (HOMA-IR) in patients with diabetes mellitus. Further investigations are needed to elucidate the causal pathways involved. Despite diabetes being a major contributor to CVD, several studies have excluded individuals with a medical history involving diabetes and CVD. Moreover, a retrospective cohort study found a correlation between the TyG index and an increased incidence of CVD in a non-diabetic population, with a median follow-up of 5.6 years [17]. A subsequent prospective cohort study corroborated this association (per 1 standard deviation, HR 1.634; 95% CI 1.304–2.047) after a 6-year follow-up period, again excluding patients with a documented history of diabetes mellitus and CVD [40]. Park also highlighted the significance of the TyG index for the incidence of coronary artery disease (CAD) in a nondiabetic population, reporting an HR of 2.29 (95% CI 1.50–3.51) [41].

## 5. Limitations

This study has several limitations.

First, owing to the observational design, our findings are limited to demonstrating associations rather than establishing causality between TyG index trajectories and mortality in older adults. Thus, large-scale randomized controlled trials are needed to determine causal effects.

Second, we adopted a complete-case analysis for longitudinal TyG measurements. If missingness was non-random (*e.g.*, participants with deteriorating metabolic health were more likely to be lost to follow-up), our approach may have underestimated the true extent of health deterioration, potentially biasing results toward healthier individuals. Third, while changes in the TyG index were modeled as a time-dependent variable, other time-varying factors, such as smoking status, alcohol consumption, and physical activity, were not modeled as such. Due to limited changes in lifestyle factors and the complexity of modeling, we chose to use only baseline values.

Fourth, the interpretation of the “moderate TyG-optimal” pattern requires caution. Several alternative explanations beyond IR alone merit careful consideration. Low TyG levels may reflect underlying malnutrition or frailty rather than genuine metabolic health. Despite rigorous adjustment for measured confounders, residual confounding from unmeasured or inadequately measured factors cannot be entirely dismissed. Key variables such as medication adherence, physical activity levels, dietary composition, socioeconomic status, and mental health were not fully captured in the analysis. In particular, concurrent use of lipid-lowering agents, antidiabetic medications, or antihypertensive drugs may have directly altered TyG values and trajectory assignments. The potential for differential medication use across risk profiles could introduce bias that standard covariate adjustment cannot fully eliminate. Additionally, despite rigorous adjustment for measured confounders, residual confounding from unmeasured or inadequately measured factors cannot be entirely dismissed. The absence of certain data precluded adjustments for socioeconomic status, which may have introduced confounding effects [42], despite our efforts to control for established risk factors for CVD mortality. The observational nature of our study also precludes the determination of the temporal precedence between TyG trajectories and mortality. Reverse causality is plausible: individuals with subclinical diseases or progressive physiological decline may exhibit altered metabolic profiles, including lower TyG, years before death, driving the apparent association without any causal role of the TyG index. Additionally, survival bias may have been present: participants who survived to older ages and remained in the surveillance program likely represent a healthier subset of the original cohort, truncating the more adverse trajectories that might have been observed with longer or more complete follow-up. This selection toward healthier survivors may have amplified the apparent mortality advantage of the “moderate TyG-optimal” group. Furthermore, the “moderate TyG-optimal” phenotype may represent a heterogeneous composite of distinct physiological states rather than a singular entity. In patients with type 2 diabetes, lean individuals with high TyG paradoxically have been reported to have a higher relative cardiovascular risk than obese counterparts, challenging BMI-based risk assumptions. Some participants classified as “moderate TyG-optimal” may have true insulin sensitivity, whereas others may have low TyG driven by nutritional insufficiency, sarcopenia, or specific medication use—subpopulations that our trajectory framework cannot distinguish. The “moderate TyG-optimal” pattern also cannot be directly extrapolated to younger or healthier populations, and unmeasured interactions with other metabolic markers (*e.g.*, inflammatory indices, adiponectin, liver enzymes) may further refine or modify the observed associations. Considering these complexities, our results should be considered hypothesis-generating, and future studies should incorporate direct measurements of nutritional markers (*e.g.*, albumin, prealbumin, MINI Nutritional Assessment), frailty indices, and body composition metrics to more precisely disentangle the mechanisms underlying U-shaped TyG-mortality relationships.

Fifth, mortality data were obtained from the NMSS via record linkage. No independent verification of deaths or cause-of-death codes was performed. Therefore, potential misclassification of all-cause and cause-specific mortality cannot be ruled out, especially for causes requiring detailed clinical information. As a routine administrative registry, the NMSS is susceptible to coding errors and incomplete documentation. These limitations should be considered when interpreting cause-specific mortality results. Sixth, while we described multiple diagnostic tests, model selection was ultimately based on case-study-appropriate model interpretation by a multidisciplinary research team [23].

Finally, because this study included only Chinese participants, external validation in other ethnic groups is required to assess the generalizability of our findings. Future work should address these limitations and further clarify the clinical utility of TyG trajectories.

## 6. Conclusions

In conclusion, this study builds on previous research by elucidating the association between TyG index trajectories and ACM in a cohort of 93,418 older adults. Our findings indicate that participants exhibiting moderate-to-low or high TyG index trajectories had higher risks of CVD and ACM. Thus, longitudinal assessment of TyG index trajectories may serve as a valuable tool for identifying individuals at elevated risk of ACM and for guiding targeted preventive and therapeutic interventions.

## Data Availability

Data are available upon reasonable request. Restrictions apply to the availability of these data.
