# Supplementary material for: Age-Related Trajectories of the Triglyceride–Glucose Index and All-Cause Mortality in Older Chinese Adults
Source: Rev Cardiovasc Med. 2026 Sep 16;27(9):50652. doi: 10.31083/RCM50652 (PMC13653179; doi:10.31083/RCM50652)
Supplement: Supplementary file 1 [file 2153-8174-27-9-50652-s1.zip › Supplementary Material.docx]

**Additional File**

**Supplementary Table 1: Baseline demographic and clinical characteristics of the final analytic cohort (n=93,418) and the original total population (n=652,314), with standardized mean differences (SMD)**

| **Characteristics** | **overall** | **total** | ***p*-value** | **SMD** |
| --- | --- | --- | --- | --- |
|  | 652,314 | 93,418 |  |  |
| **Age, years** | 72(68,77) | 71(67,75) | ＜0.001 | 0.129 |
| **Male, n(%) =1** | 281,930(43.22) | 32,435(34.72) | ＜0.001 | 0.183 |
| **Smoking, n(%)** |  |  |  |  |
| **no** | 550,226(84.35) | 82,276(88.07) | ＜0.001 | 0.111 |
| **current** | 102,088(15.65) | 11,142(11.93) |  |  |
| **Drinking, n(%)** |  |  |  |  |
| **no** | 582,190(89.25) | 86,023(92.08) | ＜0.001 | 0.097 |
| **current** | 70,124(10.75) | 7395(7.92) |  |  |
|  |  |  |  |  |
| **BMI, kg/m2** | 22.83(21.01,24.84) | 24.06(21.97,25.97) | ＜0.001 | 0.256 |
| **WC, cm** | 80(75,85) | 82(76,88) | ＜0.001 | 0.2 |
| **eGFR, ml/min per 1.73 m2** | 74.76(60.31,94.36) | 91.45(59.86,93.85) | ＜0.001 | 0.105 |

Specifically, we followed a multi-step decision process that encompasses:

Step 1: Model specification. Serial LCTMs with 2 to 5 classes were fitted, testing linear and quadratic trajectory shapes.

Step 2: Statistical criteria. The optimal number of trajectory groups was jointly determined using (i) Bayesian Information Criterion (BIC)—the model with the lowest BIC was prioritized; (ii) Akaike Information Criterion (AIC)—considered alongside BIC; (iii) average posterior probability (AvePP) —we required an AvePP ≥ 0.7 for each trajectory class, indicating good classification accuracy; and (iv) entropy (range: 0 to 1)—models with higher entropy (≥0.7) were preferred, reflecting greater class separation.

**Supplementary Table 2:** **Model fit statistics and classification accuracy for trajectory solutions: Bayesian Information Criterion (BIC), Akaike Information Criterion (AIC), average posterior probability (AvePP), and entropy**

Step 3: Model adequacy. Following the framework by Lennon et al., after selecting the candidate model based on fit statistics, we further assessed model adequacy using discrimination tools (degree of separation between classes and examination of residual plots) to ensure that the trajectories were well-differentiated. The average posterior probabilities (APPA) for each trajectory group were all above 0.7 (Group1: 0.762, Group2: 0.776, Group3: 0.837, Group4: 0.851), indicating excellent classification accuracy. Mahalanobis distances were also computed but, due to the near‑singular within‑group covariance matrices and the high‑dimensional scaling, the distances were not informative (all groups showed a mean self‑distance of approximately 2.0). We therefore rely on the more robust envelope and APPA diagnostics, which are standard in latent class trajectory modeling (Lennon et al., 2018).

**Supplementary Fig. 1: Elsensohn's envelope of residuals: mean residuals by age and trajectory group with 95% confidence intervals**

Step 4: Clinical plausibility. The final model was also evaluated on clinical grounds, i.e., whether the identified trajectory groups corresponded to biologically and clinically meaningful patterns of insulin resistance progression. This combined statistical-clinical approach has been adopted in the literature and is detailed in the revised Methods section.

**Supplementary Table 3: Baseline characteristics of participants in the quartile range of the TyG index**

| **Characteristics** | **total** | **Q1** | **Q2** | **Q3** | **Q4** | ***p*-value** |
| --- | --- | --- | --- | --- | --- | --- |
| **pAge, years** | 71.35±5.07 | 71.60±5.24 | 71.49±5.12 | 71.40±5.06 | 70.92±4.83 | <0.001 |
| **Male, n(%) =1** | 32,435(34.72) | 8,906(38.11) | 8,426(36.10) | 7,877(33.71) | 7,226(30.96) | <0.001 |
| **Smoking, n(%)** |  |  |  |  |  |  |
| **1（never）** | 82,276(88.07) | 20,531(87.84) | 20,616(88.33) | 20,593(88.12) | 20,536(88.00) | 0.776 |
| **2（former）** | 2,477(2.65) | 627(2.68) | 602(2.58) | 630(2.70) | 618(2.65) |  |
| **3（current）** | 8,665(9.28) | 2,214(9.47) | 2,122(9.09) | 2,146(9.18) | 2,183(9.35) |  |
| **Drinking, n(%)** |  |  |  |  |  |  |
| **1（never）** | 86,023(92.08) | 21,439(91.73) | 21,533(92.26) | 21,549(92.21) | 21,502(92.14) | 0.001 |
| **2（occasionally）** | 4,329(4.63) | 1,096(4.69) | 1,049(4.49) | 1,100(4.71) | 1,084(4.64) |  |
| **3（always）** | 1,252(1.34) | 364(1.56) | 337(1.44) | 290(1.24) | 261(1.12) |  |
| **4（everyday）** | 1,814(1.94) | 473(2.02) | 421(1.80) | 430(1.84) | 490(2.10) |  |
| **Regular exercise, n(%)** |  |  |  |  |  |  |
| **1（everyday）** | 24,351(26.13) | 5,168(22.18) | 5,447(23.39) | 6,561(28.13) | 7,175(30.80) | <0.001 |
| **2（≥1 times/week）** | 4,712(5.06) | 1,270(5.45) | 1,214(5.21) | 1,085(4.65) | 1,143(4.91) |  |
| **3（occasionally）** | 10,372(11.13) | 2,584(11.09) | 2,704(11.61) | 2,680(11.49) | 2,404(10.32) |  |
| **4（never）** | 53,774(57.69) | 14,277(61.28) | 13,927(59.79) | 12,995(55.72) | 12,575(53.98) |  |
| **BMI, kg/m^2^** | 24.06±3.09 | 23.52±3.01 | 23.76±2.99 | 24.26±3.11 | 24.72±3.11 | <0.001 |
| **WC, cm** | 82.35±9.41 | 80.53±9.25 | 81.41±9.20 | 83.01±9.32 | 84.46±9.38 | <0.001 |
| **SBP, mmHg** | 138.25±17.00 | 135.17±15.86 | 135.87±15.91 | 139.36±17.08 | 142.59±18.03 | <0.001 |
| **DBP, mmHg** | 81.72±9.27 | 80.60±8.73 | 81.10±8.65 | 82.12±9.42 | 83.04±10.03 | <0.001 |
| **Fasting glucose, mmol/l** | 6.77±2.62 | 5.36±1.07 | 5.88±1.16 | 6.83±1.72 | 9.02±3.76 | <0.001 |
| **Total cholesterol, mmol/l** | 4.79±3.10 | 4.25±1.81 | 4.46±1.85 | 4.73±1.61 | 5.72±5.28 | <0.001 |
| **Triglycerides, mmol/l** | 1.87±1.97 | 0.98±0.26 | 1.37±0.24 | 1.70±0.39 | 3.42±3.42 | <0.001 |
| **LDL, mmol/l** | 2.57±0.91 | 2.38±0.82 | 2.52±0.83 | 2.67±0.90 | 2.73±1.04 | <0.001 |
| **HDL, mmol/l** | 1.50±0.83 | 1.53±0.96 | 1.52±0.85 | 1.49±0.78 | 1.43±0.73 | <0.001 |
| **Serum creatinine, umol/l** | 82.57±25.55 | 81.88±24.69 | 82.34±23.58 | 82.46±25.38 | 83.59±28.29 | 0.011 |
| **eGFR, ml/min per 1.73 m^2^** | 91.45±155.74 | 92.69±155.79 | 91.13±156.46 | 91.24±155.23 | 90.75±155.47 | <0.001 |
| **Hypertension, n(%)** | 70,077(75.01) | 16,033(68.60) | 16,441(70.44) | 18,203(77.89) | 19,400(83.13) | <0.001 |
| **Diabetes, n(%)** | 47,291(50.62) | 7,050(30.16) | 8,983(38.49) | 13,361(57.17) | 17,897(76.69) | <0.001 |
| **Dyslipidemia, n(%)** | 27,806(29.77) | 2,784(11.91) | 2,900(12.43) | 5,341(22.86) | 16,781(71.91) | <0.001 |
| **Anti-hypertensive medication, n(%)** | 65,638(70.26) | 16,094(68.86) | 16,263 (69.68) | 16,643(71.22) | 16,638(71.29) | <0.001 |
| **Hypoglycemic medication, n(%)** | 12,839(13.74) | 2,645(11.32) | 2,939(12.59) | 3,469(14.84) | 3,786(16.22) | <0.001 |

Continuous data presented as median (IQR).

Categorical data presented as number (%).

BMI, body mass index; WC, waist circumference; SBP, systolic blood pressure; DBP, diastolic blood pressure; TC, total cholesterol; TG, triglycerides; LDL, low-density lipoprotein; HDL, high-density lipoprotein; eGFR, estimated glomerular filtration rate.

**Supplementary Table 4:** **Hazard ratios (95% confidence intervals) of all-cause mortality by baseline TyG index**

| TyG index | Quartile 1 | Quartile 2 | Quartile 3 | Quartile 4 | Per 1 SD |
| --- | --- | --- | --- | --- | --- |
| all-cause mortality(N) | 1826 | 1815 | 1798 | 1925 |  |
| Unadjusted  HR (95% CI) | Reference | 1.008(0.944-1.075) | 1.017(0.953-1.086) | 1.087(1.019-1.158) | 1.023(0.990-1.057) |
| P value |  | 0.814 | 0.605 | 0.011 | 0.182 |
| p for trend | 0.013 | | | |  |
| Model 1  HR (95% CI) | Reference | 1.030(0.965-1.099) | 1.063(0.996-1.135) | 1.213(1.138-1.294) | 1.079(1.045-1.114) |
| P value |  | 0.370 | 0.064 | 0.000 | 0.000 |
| p for trend | 0.000 | | | |  |
| Model 2  HR (95% CI) | Reference | 1.034(0.969-1.104) | 1.079(1.011-1.151) | 1.238(1.161-1.320) | 1.088(1.054-1.123) |
| P value |  | 0.312 | 0.023 | 0.000 | 0.000 |
| p for trend | 0.000 | | | |  |
| Model 3  HR (95% CI) | Reference | 1.044(0.978-1.115) | 1.110(1.039-1.186) | 1.321(1.235-1.414) | 1.189(1.144-1.235) |
| P value |  | 0.192 | 0.002 | 0.000 | 0.000 |
| p for trend | 0.000 | | | |  |
| Model 4  HR (95% CI) | Reference | 1.039(0.973-1.109) | 1.108(1.037-1.184) | 1.359(1.262-1.464) | 1.129(1.088-1.171) |
| P value |  | 0.252 | 0.002 | 0.000 | 0.000 |
| p for trend | 0.000 | | | |  |

Model 1: Adjusted for age and sex;

Model 2: Adjusted for age, sex, smoking, drinking and exercise.

Model 3: Adjusted for age, sex, smoking, drinking, exercise, BMI, WC, serum creatinine, eGFR, SBP, DBP, TC, LDL and HDL.

Model 4: Adjusted for age, sex, smoking, drinking, exercise, BMI, WC, serum creatinine, eGFR, hypertension and dyslipidemia.

TyG index, triglyceride-glucose index; HR, hazard ratio; CI, confidence interval; SD, standard deviation

**Supplementary****Fig. 2: Hazard ratios (95% confidence intervals) of all-cause mortality by baseline TyG index**

**Supplementary Table 5: Hazard ratios (95% confidence intervals) of all-cause mortality by trajectory groups of TyG index**

| TyG index trajectories | Trajectory 3 | Trajectory 1 | Trajectory 2 | Trajectory 4 |
| --- | --- | --- | --- | --- |
| all-cause mortality(N) |  |  |  |  |
| Unadjusted  HR (95% CI) | Reference | 1.204(1.058-1.371) | 1.243(1.180-1.309) | 1.204(1.104-1.313) |
| *p* value |  | 0.005 | 0.000 | 0.000 |
| *p* for trend | 0.324 | | | |
| Model 1  HR (95% CI) | Reference | 1.043(0.915-1.188) | 1.133(1.075-1.194) | 1.234(1.132-1.346) |
| *p* value |  | 0.528 | 0.000 | 0.000 |
| *p* for trend | 0.087 | | | |
| Model 2  HR (95% CI) | Reference | 1.039(0.912-1.184) | 1.126(1.068-1.186) | 1.237(1.135-1.350) |
| *p* value |  | 0.564 | 0.000 | 0.000 |
| *p* for trend | 0.069 | | | |
| Model 3  HR (95% CI) | Reference | 0.995(0.873-1.135) | 1.105(1.047-1.165) | 1.247(1.143-1.360) |
| *p* value |  | 0.942 | 0.000 | 0.000 |
| *p* for trend | 0.011 | | | |
| Model 4  HR (95% CI) | Reference | 0.998(0.875-1.138) | 1.104(1.047-1.165) | 1.243(1.139-1.357) |
| *p* value |  | 0.972 | 0.000 | 0.000 |
| *p* for trend | 0.014 | | | |

**Supplementary Table 6: Hazard ratios (95% confidence intervals) of all-cause mortality by trajectory groups of TyG index in different sensitivity analyses.**

| TyG index trajectories | Trajectory 3 | Trajectory 1 | Trajectory 2 | Trajectory 4 |
| --- | --- | --- | --- | --- |
| Sensitivity analyses 1  HR (95% CI) | Reference | 0.981(0.859-1.121) | 1.098(1.040-1.158) | 1.251(1.146-1.365) |
| *p* value |  | 0.780 | 0.001 | 0.000 |
| Sensitivity analyses 2  HR (95% CI) | Reference | 1.030(0.893-1.186) | 1.125(1.060-1.193) | 1.255(1.139-1.383) |
| *p* value |  | 0.688 | 0.000 | 0.000 |
| Sensitivity analyses 3  HR (95% CI) | Reference | 1.058(0.903-1.240) | 1.205(1.118-1.298) | 1.346(1.161-1.561) |
| *p* value |  | 0.483 | 0.000 | 0.000 |

Sensitivity Analysis 1: Delete deaths within 2 years (Model 3)

Sensitivity analysis 2: hypoglycemic drug users were excluded

Sensitivity analysis 3: Those with FBG≥7.0 and TG≥1.7 at baseline were excluded
